# Micronutrient intakes of lactating mothers and their association with breast milk concentrations and micronutrient adequacy of exclusively breastfed Indonesian infants

**DOI:** 10.1093/ajcn/nqz047

**Published:** 2019-06-01

**Authors:** Lisa Daniels, Rosalind S Gibson, Aly Diana, Jillian J Haszard, Sofa Rahmannia, Dimas E Luftimas, Daniela Hampel, Setareh Shahab-Ferdows, Malcolm Reid, Larisse Melo, Yvonne Lamers, Lindsay H Allen, Lisa A Houghton

**Affiliations:** 1 Departments of Human Nutrition; 2 Chemistry, University of Otago, Dunedin, New Zealand; 3 Faculty of Medicine, Universitas Padjadjaran, West Java, Indonesia; 4 USDA/ARS Western Human Nutrition Research Center, Davis, CA; 5 Department of Nutrition, University of California, Davis, CA; 6 Faculty of Land and Food Systems, University of British Columbia, Vancouver, Canada; 7 British Columbia Children's Hospital Research Institute, Vancouver, Canada

**Keywords:** breast-milk intake, breast-milk volume, breast-milk micronutrient concentrations, exclusively breastfed infants, maternal micronutrient intakes

## Abstract

**Background:**

Breast milk is the sole source of nutrition for exclusively breastfed infants in the first 6 mo of life, yet few studies have measured micronutrient concentrations in breast milk in light of maternal diet and subsequent infant micronutrient intakes.

**Objectives:**

We evaluated the adequacy of micronutrient intakes of exclusively breastfed Indonesian infants by measuring milk volume and micronutrient concentrations and assessed maternal micronutrient intakes and their relationship with milk concentrations.

**Methods:**

Mother–infant (2–5.3 mo) dyads (*n* = 113) were recruited for this cross-sectional study. Volume of breast-milk intake via the deuterium dose-to-mother technique over 14 d and analyzed micronutrient concentrations were used to calculate micronutrient intakes of exclusively breastfed infants. Maternal 3-d weighed food records were collected to assess median (IQR) micronutrient intakes. Multivariate regression analyses examined the association of usual maternal micronutrient intakes with milk micronutrient concentrations after adjustment for confounding variables.

**Results:**

Mean ± SD intake of breast-milk volume was 787 ± 148 mL/d. Median daily infant intakes of iron, zinc, selenium, magnesium, sodium, and B-vitamins (thiamin, riboflavin, niacin, pantothenic acid, B-6, and B-12) were below their respective Adequate Intakes. Inadequacies in maternal intakes (as % < estimated average requirements) were >40% for calcium, niacin, and vitamins A, B-6, and B-12. Significant positive associations existed between maternal usual intakes of vitamin A, niacin and riboflavin and milk retinol, nicotinamide, and free riboflavin concentrations in both unadjusted and adjusted (for infant age, milk volume, and parity) analyses (all *P* < 0.05).

**Conclusions:**

The majority of micronutrient intakes for these exclusively breastfed infants and their mothers fell below recommendations, with associations between maternal intakes and breast-milk concentrations for 3 nutrients. Data on nutrient requirements of exclusively breastfed infants are limited, and a better understanding of the influence of maternal nutritional status on milk nutrient concentrations and its impact on the breastfed infant is needed.

## Introduction

Breast milk is the sole nutritional source for infants who are exclusively breastfed, a practice recommended by the WHO until 6 mo of age. Exclusive breastfeeding is known to protect against gastrointestinal infections during early infancy, especially in low- and middle-income countries, and to support optimal growth and development (1). Indonesia is a lower-middle-income country with relatively high rates of exclusive breastfeeding (∼41%) (2); however, the adequacy of nutrient intakes of exclusively breastfed Indonesian infants is unknown.

Several studies have measured micronutrient concentrations in breast milk, although few have quantified both the volume and micronutrient concentrations of breast milk consumed simultaneously with the use of state-of-the-art techniques (3). In the past, instead of employing isotope tracer methods to measure the volume of breast milk consumed unobserved, test-weighing and maternal breast-milk expression were used, approaches with the potential to interfere with physiologic or behavioral aspects of lactation (4). In addition, the accuracy and precision of micronutrient concentrations in breast milk, most notably for the vitamins, has been limited by the absence of analytic procedures capable of quantifying the different vitamer forms and their contribution to total milk vitamin content (5, 6). Furthermore, none of these studies have also measured concurrently maternal dietary intakes. Consequently, whether micronutrient intakes of exclusively breastfed infants derived from these measurements have been compromised by deficits in maternal intakes has rarely been investigated, despite reports of chronic deficits in micronutrient intakes among women in low-income (7, 8) and lower-middle-income countries such as Indonesia (9).

Therefore, the primary objective of this study was to assess the adequacy of micronutrient intakes of exclusively breastfed Indonesian infants by measuring milk volume and micronutrient concentrations through the use of state-of-the-art methods. The secondary objective of the study was to determine the association of maternal diet with breast-milk micronutrient concentrations.

## Methods

### Study site and participants

This cross-sectional study was conducted in Tanjungsari, Sukasari, and Pamulihan subdistricts of Sumedang district, West Java, Indonesia between April and September 2016. Exclusively breastfeeding mother–infant dyads were purposively recruited from these 3 subdistricts of Sumedang by community health workers (cadres). Inclusion criteria for infants were: aged from 2 to 5.5 mo, born full-term (>37 wk gestation), ≥2500 g at birth, apparently healthy with no evidence of chronic disease or acute malnutrition, and had been exclusively breastfed until the day of recruitment. With a sample size of *n* = 110, we were able to determine estimates of means with a 95% precision interval of at least ±0.2SD. Furthermore, regression models were constructed with a number of covariates (based on the rule of thumb of ≥10 observations per predictor).

Ethical approval was obtained from the Human Ethics Committees of Padjadjaran University, Indonesia, and the University of Otago, New Zealand. Informed written consent to participate was given by the parents. Participants were free to withdraw from the study at any time.

### Sociodemographic, health, and breastfeeding status

Pretested questionnaires on sociodemographic status, infant morbidity (occurrence of diarrhea, vomiting, fever, or cough, or a combination of these, during the 2 wk of data collection), and breastfeeding practices at baseline were administered to mothers by trained research assistants. On 6 nonconsecutive days cadres observed daytime breastfeeding practices in each household from 0600 to 1800, with nighttime breastfeeding practices assessed by maternal recalls. On nonobservation days, mothers were requested to continue to comply to only give breast milk to their participating infant. To ensure the inclusion of exclusively breastfed infants only, details of feeding practices (including the frequency and amount of any fluid, including water, and accidental food consumption) were collected by research assistants or cadres based on 24-h maternal recalls. In addition, unannounced spot-checks were also conducted by cadres to check on the compliance with exclusive breastfeeding.

### Anthropometry

Weight (minimal clothing) and height of mothers, and weight (nude), recumbent length, head circumference, and mid-upper arm circumference (MUAC) of infants were measured at baseline with the use of calibrated equipment and standardized techniques, as described previously (10). Both infant and maternal weight were measured again on day 14. Anthropometric measurements were recorded in duplicate, or triplicate if the difference between the first and second measurement exceeded the maximal allowable difference (i.e., 100 g for weight, 7.0 mm for length, 5.0 mm head circumference, 5.0 mm MUAC) (11). Infants’ length-for-age, weight-for-age, weight-for-length, head circumference-for-age, and MUAC) (for infants aged >3 mo) *z* scores were calculated with WHO AnthroPlus 3.2.2 (12). Maternal BMI was also calculated.

### Measurement of breast-milk volume

Infant intake of breast-milk volume was measured over a 14-d period through the use of the dose-to-mother deuterium-dilution method (13). Briefly, on day 0 (after assurance that neither mother or infant had consumed food or fluid in the past 30 min), predose saliva samples were collected from both mother (2 mL) and infant (0.5 mL), after which mothers received an accurately measured oral dose of 30 g of diluted deuterium oxide solution (^2^H_2_O) (Sigma-Aldrich) accurately measured to the nearest 0.01 g and diluted in ∼50 g drinking water.

Postdose saliva samples were collected from the mother and infant on days 1, 2, 3, 5, 6, 13, and 14 (day 6 was for infants >3 mo only). For the saliva collection, a sterile cotton ball was inserted into the mouth, and after a few minutes, the saliva was expressed from the wet cotton ball into sterile tubes with the use of a 10-mL disposable syringe. All saliva samples were stored at −20°C until analysis of deuterium enrichment by Fourier transform infrared spectrometry (13). The deuterium enrichment in each saliva sample was assessed until the precision estimate was <0.3 ppm. There were no reported side effects from the administration of deuterium oxide.

Breast-milk volume was calculated with the use of the equations and methods described by the International Atomic Energy Agency (13). Briefly, fitted curves were generated in Microsoft Excel (2013) with data from the saliva samples and timing of administration of the deuterium dose to the mother.

### Collection and micronutrient analysis of breast-milk samples

Full breast-milk samples were collected in the mornings on day 14 for micronutrient analysis. Mothers were requested to refrain from applying creams or powders to their breasts and from breastfeeding on 1 breast at least 2 h prior to the scheduled breast-milk collection. After washing their hands and the selected breast with distilled, deionized water to prevent any adventitious contamination, mothers expressed breast-milk samples either manually or via a breast pump until the breast was empty (to allow for the collection of both fore- and hind-milk) into acid-washed glass bottles. Two aliquots of 1 mL (for micronutrient analysis) were transferred with the use of trace-element free techniques into acid-washed microtubes, and stored at −80°C until analysis.

Major elements (sodium, magnesium, phosphorus, potassium, and calcium) and trace elements (iron, copper, zinc, and selenium) were analyzed in the Centre for Trace Element Analysis, Department of Chemistry, University of Otago, New Zealand, by inductively coupled plasma mass spectrophotometry (Agilent 7900; Agilent Technologies). Briefly, 10 mL quartz subboiling 14 N nitric acid and 1 mL of 30% hydrogen peroxide were added to ∼1 g of the breast-milk sample. Samples were then microwave digested at 200°C for 15 min and dried down to ∼1 mL. The final sample was diluted to 25 mL with 2% aqueous nitric acid. The precision and accuracy were checked with the use of in-house pooled samples and multielement reference standards (SRM 1846, infant formula) from the National Institute of Standards and Technology, presented in **Supplemental Table 1**.

Vitamins were analyzed in the laboratory of LHA (Western Human Nutrition Research Centre, UC Davis, CA), except for cobalamin which was analyzed in the laboratory of YL (University of British Columbia, Vancouver, Canada). Free thiamin, thiamin monophosphate (TMP), and thiamin pyrophosphate (TPP) were analyzed by HPLC with fluorescence detection performed with an Agilent 1200 series HPLC by precolumn derivatization into the thiochrome esterase previously described (5). Total thiamin was calculated based on the measured concentrations for each vitamer and expressed as free thiamin. Riboflavin, flavin adenine dinucleotide (FAD), nicotinamide, pyridoxal, pyridoxine, biotin, and pantothenic acid were analyzed by ultra-performance liquid chromatography tandem mass-spectrometry (Hampel et al., in preparation) with a Waters ACQUITY UPLC system coupled with an Sciex 4000 QTRAP mass spectrometer. Cobalamin was analyzed by competitive chemiluminescent enzyme immunoassay (IMMULITE 1000; Siemens) (14), and retinol, β-carotene, α-tocopherol, and γ-tocopherol were determined by HPLC with diode array detection (Agilent 1200 series) as previously described (15) (see Supplemental Table 1).

Individual breast-milk fat concentration was determined through the use of the creamatocrit method (CreamatocritPlus; EKF Diagnostics) (16) in the Pharmacology Laboratory, Universitas Padjadjaran, Bandung, Indonesia (CV = 6.9%). Breast-milk fat concentration was used to adjust fat-soluble vitamins A and E, as fat is known to influence their concentrations in breast milk (17, 18).

### Assessment of infant micronutrient intakes from breast milk

Measurement of the breast-milk volume consumed by each infant was used to estimate the average daily micronutrient intakes of exclusively breastfed infants, calculated as analyzed micronutrient concentration (mg/L or μg/L) multiplied by the individual breast-milk volume (L). Vitamins A, E, and B-6, total thiamin, and total riboflavin were calculated as follows: vitamin A [as retinol equivalents (REs), where 1 μg retinol = 1 RE and 6 μg β-carotene = 1 RE] (19); vitamin E [as tocopherol equivalents (TEs), where 1 α-TE = 1 mg α-tocopherol] (20); vitamin B-6 (pyridoxal + pyridoxine) (19); vitamin B-1 [thiamin + (TPP × 0.707) + (TMP × 0.871)] (21); and vitamin B-2 [riboflavin + (FAD × 0.479)] (22). Vitamins A and E were also expressed as concentrations per gram of milk fat (μg/g); calculated as the individual vitamin A (retinol) and E (α-tocopherol) concentrations per volume (μg/L) divided by the individual fat concentration of breast milk (g/L) (20, 23).

Micronutrient intakes of infants were compared with corresponding Adequate Intake (AI) amounts set recently by the European Food Safety Authority (EFSA) (19) and the International Zinc Nutrition Consultative Group (IZiNCG) (24).

### Assessment of maternal micronutrient intakes

On 3 nonconsecutive observation days, cadres weighed 12-h daytime food intakes (0600–1800) consumed in the home combined with 12-h maternal recalls of the foods and beverages consumed between the hours of 1800 and 0600, to estimate total 24-h food intakes. Ingredients of mixed dishes and the amounts consumed were also weighed to determine the weight of the actual ingredients consumed by the mother. Daily intakes of energy and selected nutrients were calculated from a specially compiled Indonesian food composition table, described elsewhere (25). Micronutrient values for all wheat flour products were adjusted to reflect the government mandatory fortification concentrations (thiamin, 2.5 mg/kg; riboflavin, 4 mg/kg; iron, 50 mg/kg; zinc, 30 mg/kg; and folate, 2 mg/kg) (26).

The estimated average requirements (EARs) compiled by WHO (27) as modified by Arimond et al. (28) for lactating women were used for all micronutrients with the exception of calcium, iron, and zinc. For calcium and iron, EARs from the Institute of Medicine were used (29, 30), although iron was adjusted to reflect 10% bioavailability. For zinc, the IZiNCG EAR for mixed refined vegetarian diets was used (31).

### Statistical analysis

All variables were assessed for normality. Medians (IQR) were used to describe skewed data including micronutrient concentrations of breast milk, and daily micronutrient intakes of infants and their mothers. Intakes of pyridoxine for infants were a notable exception, because there was a high proportion with 0 values. Consequently, infant pyridoxine intakes were described as the proportion with milk concentrations >0, and the associated median (IQR). Pearson's correlation coefficients were calculated to assess the correlation between breast-milk volume and infant age and bodyweight. Regression analysis was used to assess the association between breast-milk volume per kilogram body weight and infant age, unadjusted and adjusted for sex. To determine whether morbidity influenced this association a sensitivity analysis was conducted. Student's 2-sample *t* tests were used to assess whether there were any mean differences between female and male infants for bodyweight and breast-milk volume.

The Multiple Source Method program (32) was used to produce usual intake distributions for the study population, based on estimates of the individual usual intakes. The EAR cutpoint method was then applied to the usual intake distributions to assess the prevalence of inadequate intakes (except for iron). To determine the prevalence of inadequate intakes of iron, the full probability approach applying the probabilities for female oral contraceptive users were selected (29) assuming a 60% reduction in menstrual loss as more reflective of losses during the first 6 mo of lactation.

Regression analysis was used to determine the association between breast-milk micronutrient concentrations and usual maternal micronutrient intakes, adjusted for age of the infant, breast-milk volume, and parity. Because breast-milk micronutrient concentrations were skewed, the regression analysis was conducted with the use of log-transformed breast-milk micronutrient concentrations and then expressed as a percentage difference. Regression analysis was also used to determine association between either age of infants, parity, or breast-milk volume; and breast-milk micronutrient concentrations. Residuals of all regression models were plotted and visually assessed for homogeneity of variance and normality.

All analyses were conducted with Stata version 15.1 statistical software (StataCorp LP). *P* values <0.05 were considered to be significant.

## Results

### Mother–infant dyad characteristics

Of the 121 approached, 8 mother–infant dyads were excluded because of biologically implausible breast-milk volume data (*n* = 1), or failure to comply with exclusive breastfeeding, based on home observations (*n* = 7) (**Supplemental Figure 1**). Characteristics of mothers and full-term infants are presented in **Table 1**. Mean maternal BMI was 24 kg/m^2^. Of the infants, 5% were underweight, 7% were stunted, and 1% wasted. No dietary supplements were taken by any of the mother–infant dyads during lactation.

**TABLE 1:** Characteristics of exclusively breastfed mother–infant dyads (*n* = 113)^1^

Characteristics	Mean ± SD^2^
Maternal	
Age, y	25.8 ± 6.1
Weight, kg	54.5 ± 9.6
Height, cm	150.8 ± 5.4
BMI, kg/m^2^	24.0 ± 3.8
Parity	
Primiparous	60 (53)
Multiparous	53 (47)
Occupation	
Housewife	95 (84)
Other^3^	18 (16)
Education	
Primary school^4^	44 (39)
Secondary school^4^	62 (55)
Tertiary	7 (6)
Infant	
Age, mo	3.3 ± 0.8
Sex is female	58 (51)
Morbidity during data collection	
Diarrhea	4/113 (4)
Vomiting	6/113 (5)
Fever	14/113 (12)
Cough	16/113 (14)
Anthropometry	
WAZ	−0.42 ± 0.91
WAZ (<–2 SD)	6/113 (5)
LAZ	−0.87 ± 0.81
LAZ (<–2 SD)	8/113 (7)
WLZ	0.39 ± 1.0
WLZ (<–2 SD)	1/113 (1)
HCAZ	−0.46 ± 0.83
MUAC, cm	14.4 ± 1.1
MUACZ^5^	1.04 ± 1.0

1 HCAZ, head circumference-for-age *z* score; LAZ, length-for-age *z* score; MUAC, mid-upper arm circumference; MUACZ, mid-upper arm circumference-for-age *z* score; WAZ, weight-for-age *z* score; WLZ, weight-for-length *z* score.

2 Values are means ± SDs, or *n* (%).

3 Other work: open stall at home (*n* = 7), making crafts at home (*n* = 8), teacher (*n* = 2), agricultural worker (*n* = 1).

4 Includes those who did not graduate: primary school (*n* = 2); secondary school (*n* = 1).

5 Only calculated for infants >3 mo of age (*n* = 66).

### Breast-milk intakes

**FIGURE 1:**
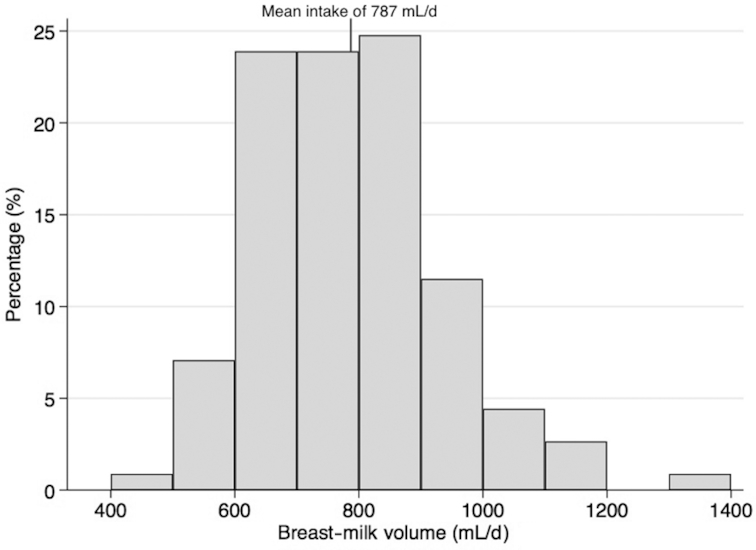
Breast-milk intake of exclusively breastfed infants aged 2–5.3 mo (*n* = 113).

**FIGURE 2:**
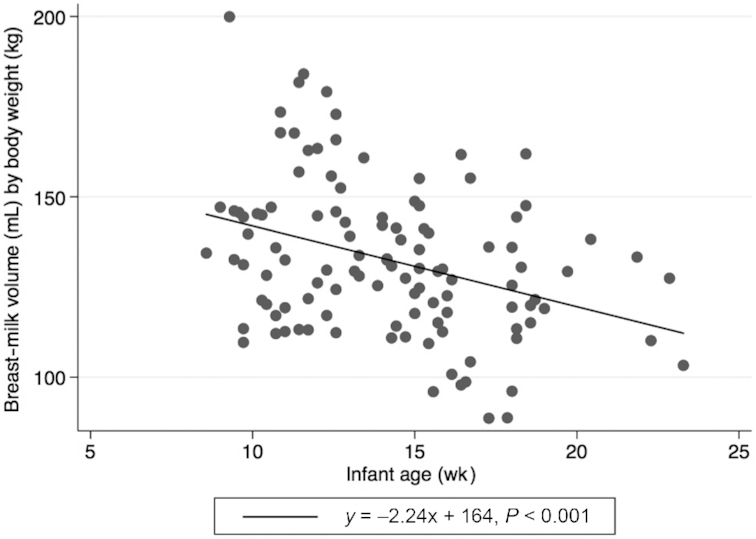
Association between breast-milk intake per kg of body weight and infant age (*n* = 113). The estimated line from regression of breast-milk volume per kg of body weight (mL) = −2.24 × [infant age (wk)] + 164.

**Figure 1** presents the distribution of breast-milk intakes. The mean ± SD intake of breast milk was 787 ± 148 mL/d. Breast-milk volume was not associated with infant age (*r* = 0.09, *P* = 0.33), but was positively correlated with infant body weight (*r* = 0.54, *P* < 0.001). Hence, volume of breast milk consumed per kilogram body weight was used to describe the association between breast-milk volume (per kg body weight) and infant age (**Figure 2**). In a sensitivity analysis, when infants with morbidity were excluded, the association between breast-milk volume (per kg body weight) and infant age, although attenuated, remained significant (**Supplemental Figure 2**). When all infants were included in a regression analysis, there was a 2 mL reduction in breast-milk intake (per kg body weight) per weekly increase in infant age, which was independent of sex (β coefficient: −2.26; 95% CI: −3.41, −1.10; *P* < 0.001). Female infants were 0.5 kg lighter than male infants (*P* < 0.001) and consumed 55 mL of breast milk per day less than male infants (*P* = 0.049).

### Infant intakes of fat and micronutrients from breast milk

The median breast-milk fat concentration was 41.8 g/L (IQR: 31.5–50.1 g/L). Taking into account breast-milk volume, the median daily intake of fat from breast milk was 33.0 g/d (IQR: 23.6–41.9 g/d). The median concentration of vitamin A (retinol) per gram of milk fat was 13.3 μg/g (IQR: 9.7–18.6 μg/g) and the corresponding value for vitamin E was 150 μg/g (IQR: 106–192 μg/g).

Daily infant micronutrient intakes from breast milk are presented in **Table 2**. Of the major elements and trace elements analyzed, median daily intakes of sodium, magnesium, iron, copper, zinc, and selenium from breast milk were below their respective AIs set by EFSA, and below the AI for zinc set by IZiNCG. Median phosphorus intakes were equal to the AI, whereas the median intake of calcium was greater than the AI. Breast-milk intakes of thiamin, riboflavin, niacin (as nicotinamide only), pantothenic acid, vitamin B-6, and vitamin B-12 (as cobalamin) were all below the AIs set by EFSA, whereas those for vitamins A, E, and biotin were greater than the AIs (Table 2). Median (IQR) breast-milk concentrations (Table 2) were compared with literature data by country and lactation period (**Supplemental Table 2**).

**TABLE 2:** Breast-milk volume, micronutrient concentration, and daily intake of exclusively breastfed infants (2–5.3 mo of age)^1^

		Breast-milk volume (*n* =113)	
	Median breast-milk concentration (IQR)^2^	Median daily intake from breast milk (IQR)^2^	EFSA (2013) AI
Micronutrient	—	782 (682–879) mL/d	800 mL/d
concentrations^3^			
Major elements (*n* = 111)			
Sodium	122 (101–193) mg/L	102 (76.3–148) mg/d	120 mg/d
Magnesium	30.1 (26.3–35.9) mg/L	24.1 (18.9–30.5) mg/d	25 mg/d
Phosphorus	128 (119–145) mg/L	100 (84.5–121) mg/d	100 mg/d
Potassium	435 (402–499) mg/L	346 (294–419) mg/d	400 mg/d
Calcium	272 (247–300) mg/L	206 (182–254)^4^ mg/d	200 mg/d
Trace elements (*n* = 111)			
Iron	0.22 (0.15–0.33) mg/L	0.17 (0.12–0.25) mg/d	0.3 mg/d
Copper	0.26 (0.21–0.32) mg/L	0.21 (0.16–0.26) mg/d	0.3 mg/d
Zinc	0.99 (0.72–1.3) mg/L	0.79 (0.50–1.03) mg/d	2^5^mg/d
Selenium	10.2 (8.7–12.9) μg/L	8.1 (6.7–10.3)^4^μg/d	12.5 μg/d
Vitamins (*n* = 112)			
Retinol	544 (391–759) μg/L	425 (276–639) μg/d	
β-Carotene	35.5 (30.8–46.3) μg/L	27.4 (22.8–38.9) μg/d	
Vitamin A	598 (431–805) μg/L	429 (279–664)^4^ μg RE/d^6^	350 μg RE/d^6^
α-Tocopherol (E)	5,707 (4,240–7,484) μg/L	4,441 (3,265–6,602) μg/d	
γ-Tocopherol (E)	1,669 (1,266–2,061) μg/L	1,264 (914–1,614) μg/d	
Vitamin E^7^	5.7(4.2–7.5) mg α-TE/L	4.4 (3.3–6.1) mg α-TE/d	3 mg α-TE/d
TMP	76.4 (58.9–93.7) μg/L	59.0 (45.1–77.9) μg/d	
TPP	2.9 (2.1–4.0) μg/L	2.2 (1.6–3.2) μg/d	
Free thiamin	20.9 (14.4–28.0) μg/L	16.1 (11.4–22.0) μg/d	
Total thiamin (B1)^8^	99.4 (83.9–121) μg/L	0.08 (0.06–0.10)^4^ mg/d	0.2 mg/d
Free riboflavin	8.6 (2.0–17.2) μg/L	7.2 (1.3–13.7) μg/d	
FAD	128 (99.5–167) μg/L	106 (73.9–132) μg/d	
Total riboflavin (B-2)^9^	75.2 (57.7–97.0) μg/L	0.06 (0.05–0.08)^4^ mg/d	0.3 mg/d
Nicotinamide (B-3)^10^	378 (227–590) μg/L	0.29 (0.19–0.45)^4^ mg/d	2^11^ mg/d
Pantothenic acid (B-5)	1,540 (1,175–1,906) μg/L	1.2 (0.90–1.5) mg/d	2 mg/d
Pyridoxal (B-6)	63.8 (46.1–78.7) μg/L	50.0 (34.9–62.2) μg/d	
Pyridoxine (B-6)^12^	0.46 (0.15–1.3) μg/L	0.34 (0.11–0.95) μg/d	
Total B-6^13^	63.8 (46.7–78.7) μg/L	0.05 (0.03–0.06)^4^mg/d	0.1 mg/d
Biotin (B-7)	18.2 (17.2–19.4) μg/L	14.4 (12.1–16.5) μg/d	4 μg/d
Cobalamin (B-12)	0.28 (0.24–0.34) μg/L	0.22 (0.18–0.28)^4^μg/d	0.4 μg/d

1 AI, Adequate Intake; EFSA, European Food Safety Authority; FAD, flavin adenine dinucleotide; IZiNCG, International Zinc Nutrition Consultative Group; RE, retinol equivalent; TE, tocopherol equivalent; TMP, thiamin monophosphate; TPP, thiamin pyrophosphate.

2 Values are medians (IQRs), unless otherwise stated.

3 Median micronutrient concentration of breast milk based on individual daily breast-milk intakes, *n* = 113.

4 Micronutrients known to be affected by maternal nutritional status (33).

5 Also the value recommended by the IZiNCG for breastfed infants aged 0–5 mo (24).

6 Vitamin A as REs, where 1 RE = 6 μg β-carotene or 1 μg retinol (34).

7 Vitamin E as TEs where 1 α-TE = 1 mg α-tocopherol (20).

8 Total thiamin = thiamin + (thiamin pyrophosphate × 0.707) + (thiamin monophosphate × 0.871) (21).

9 Total riboflavin = riboflavin + (FAD × 0.479) (22).

10 *n* = 111, 1 outlier excluded.

11 Value based on niacin equivalents (19).

12 Pyridoxine was dichotomized [due to 71% (*n* = 80) of breast-milk concentrations at 0 μg/L] and therefore these values are based on those with pyridoxine concentrations >0 μg/L (*n* = 32, 29%).

13 Vitamin B-6 = pyridoxal + pyridoxine (19).

### Maternal micronutrient intakes and breast-milk micronutrient concentrations

Median (IQR) maternal usual daily micronutrient intakes are presented in **Table 3**. The prevalence of inadequacy was highest (>40%) for calcium, vitamin A, niacin, vitamin B-6, and vitamin B-12, all micronutrients provided by dietary sources alone (Table 3).

**TABLE 3:** Maternal daily micronutrient intakes, prevalence of inadequacy, and the association with breast-milk micronutrient concentrations^1^

				% change in breast-milk concentration (95% CI)^2^	
	Median (IQR)^3^	EAR^4^	Prevalence of inadequacy (%)	Unadjusted	Adjusted^5^
	*n* = 121			*n* = 111	*n* = 111
Energy, kcal/d	2211 ± 578^6^	—	—	—	—
Major elements					
Calcium, g	613 (509–750)	800^7^	81	1.1 (−17.1, 23.5)^8^	2.7 (−15.7, 25.0)^8^
Potassium, g	1.1 (0.80–1.3)	5.1^9^	—	4.7 (−3.6, 13.7)	4.7 (−4.1, 14.2)
Trace elements					
Iron, mg	18.3 (12.5–23.0)	11.7	2.6^10^	0.21 (−1.0, 1.5)	0.15 (−1.1, 1.4)
Zinc, mg	12.8 (10.8–15.0)	7^11^	1.7	0.33 (−2.1, 2.8)	0.19 (−2.3, 2.8)
Vitamins^12^					
Retinol, μg	—	—	—	24.7 (1.4, 53.5)^13^*	23.8 (0.45, 52.5)^13^*
Vitamin A, mg RAE	501 (319–841)	450	44.6	24.4 (1.9, 51.7)^14^*	23.5 (1.2, 50.7)^14*^
Thiamin (B-1), mg	1.4 (0.98–1.8)	1.2	39.7	3.4 (−9.0, 17.6)	5.0 (−7.4, 19.1)
Riboflavin (B-2), mg^15^	1.7 (1.2–2.2)	1.3	30.6	74.9 (18.8, 157)*	79.1 (21.2, 165)*
Niacin (B-3), mg^16^	12.8 (10.1–15.5)	13	55.4	4.0 (0.52, 7.6)*	4.0 (0.49, 7.6)*
Vitamin B-6, mg	1.3 (1.0–1.7)	1.7	74.4	−1.6 (−21.4, 23.2)	−5.2 (−25.1, 19.9)
Cobalamin (B-12), μg	2.5 (1.8–3.1)	2.4	47.9	3.0 (−4.3, 10.9)	3.1 (−4.3, 11.2)

1 Maternal intakes collected with the use of 3-d 12-h weighed diet records and 12-h diet recall. *Indicates *P* < 0.05. AI, Adequate Intake; EAR, estimated average requirement; IOM, Institute of Medicine; IZiNCG, International Zinc Nutrition Consultative Group; RAE, retinol activity equivalent.

2 Regression analysis where values represent a percentage change in the micronutrient breast-milk concentration for a unit increase in usual maternal micronutrient intake.

3 Usual intakes were used to determine median intakes, which were calculated by the Multiple Source Method (32).

4 Values by WHO (27) as modified by Arimond et al. (28), unless otherwise stated.

5 Adjusted for infant age (mo), breast-milk volume (L/d), and parity (multiparous compared with primiparous).

6 Means ± SDs.

7 Values from the 2011 updated IOM Dietary Reference Values (30), the EARs for lactating women aged 14–18 y are higher (1100 mg) and therefore this EAR was used for these women (*n* = 6).

8 Usual maternal calcium intake units changed from mg to g.

9 No EAR value for potassium, therefore the AI is reported (35) and the prevalence of inadequacy cannot be determined.

10 The full probability approach was used for iron with the use of the IOM probabilities for female oral contraceptive users (assumes 60% reduction in menstrual loss) (29).

11 Value from IZiNCG, assuming bioavailability of mixed refined vegetarian diets (31).

12 Unadjusted and adjusted analyses of vitamins *n* = 112, except niacin where 1 outlier was excluded (*n* = 111).

13 Change in breast-milk retinol concentration for each milligram increase in maternal usual vitamin A (RAE) intake.

14 Usual maternal vitamin A (RAE) intake units changed from μg to mg.

15 Change in breast-milk free riboflavin for each milligram increase in maternal usual riboflavin (B-2) intake.

16 Change in breast-milk nicotinamide for each milligram increase in maternal usual niacin (B-3) intake.

Maternal usual daily intakes of vitamin A, niacin, and riboflavin were positively associated with breast-milk concentrations in both the unadjusted and adjusted (for infant age, breast-milk volume, and parity) analyses (both *P* values <0.05). For each unit (mg) higher maternal usual daily vitamin A intake, breast-milk retinol and total vitamin A (as RE) concentrations increased by 23.8% (95% CI: 0.45%, 52.5%; *P* = 0.045) and 23.5% (95% CI: 1.2%, 50.7%; *P* = 0.038), respectively, in the adjusted analyses. For each mg higher usual maternal niacin and riboflavin intakes, concentrations of breast-milk nicotinamide were 4.0% (95% CI: 0.49%, 7.6%; *P* = 0.025) higher and 79.1% higher for free riboflavin (95% CI: 21.2%, 165%; *P* = 0.004), respectively, in the adjusted analysis (Table 3).

No associations were found between breast-milk volume and breast-milk micronutrient concentrations (data not shown). A significant positive association was seen between the age of the infant (in months) and total vitamin B-6 concentration in breast milk (% change: 18.8%; 95% CI: 6.4%, 32.6%) and a significant inverse association between parity (multiparous relative to primiparous) and calcium breast-milk concentration (% change: −8.3%: 95% CI: −14.4%, −1.9%).

## Discussion

To our knowledge, this is the first study to report the micronutrient intakes (major minerals, trace elements, and vitamins) of exclusively breastfed infants based on measurements of breast-milk volume and micronutrient concentrations through the use of state-of-the-art methods. In addition, maternal micronutrient intakes were assessed alongside breast-milk nutrient analysis. Our infants were observed in the home for 6 of the 14 study days together with unannounced home visits, whereas most other isotope studies have relied on maternal recall to define exclusive breastfeeding (3, 36–38).

Our mean breast-milk volume (787 mL/d) is within the range of isotopically measured intakes reported globally (552–884 mL/d) for exclusively breastfed infants aged <6 mo (3, 36–40), and comparable to that adopted by both the Institute of Medicine (780 mL/d) (29) and EFSA (800 mL/d) (19) for infants aged 0–6 mo.

We found no correlation between breast-milk volume and infant age in accordance with some (41) but not all (4, 38) earlier reports. However, the majority of our infants (*n* = 64) were between 4 and 5 mo of age when breast-milk volume remains relatively constant (4). Moreover, after adjusting for infant body weight (per kg), breast-milk intakes were inversely related to infant age, a trend attributed to the marked reduction in energy demands for infant growth from 3 to 6 mo of age compared with the first 3 mo (42).

In Supplemental Table 2 we restricted our comparison of breast-milk micronutrient concentrations to studies in which mature breast milk was collected between 1 and 6 mo postpartum from mothers of term infants who were not receiving any dietary supplements during lactation. For magnesium, phosphorus, and calcium, breast-milk concentrations were within the ranges reported previously, whereas sodium was lower, possibly arising from differences in breastfeeding frequency (43) and diurnal variation (44) and not maternal sodium intakes (45). Breast-milk calcium was negatively associated with parity, and unrelated to maternal intakes even though calcium intakes were characteristically low (9).

The copper concentration in breast milk fell within the range listed in Supplemental Table 2, whereas both iron and zinc were lower than elsewhere (Supplemental Table 2) despite maternal intakes that were mostly adequate, unlike previous reports (8). Differences in the stage of lactation (46), breast-milk measurement, collection, and analysis (47–50), exposure to adventitious contamination, and for iron, maternal hemoglobin status during pregnancy and lactation (51), may have contributed to the apparent discrepancies. Our low breast-milk selenium concentrations probably reflect low maternal selenium intakes (52, 53) in view of the low regional selenium concentrations in soil (54) and reported suboptimal serum selenium concentrations in Indonesian breastfed infants (55) and lactating women (9).

Both breast-milk retinol and total vitamin A concentrations were higher than other Asia/Pacific values in Supplemental Table 2, and when expressed as per gram of milk fat, the adjusted retinol concentrations were not indicative of risk of vitamin A deficiency (i.e., >8 μg/g) among the infants (56), even though 45% of the lactating mothers had inadequate intakes. Moreover, higher maternal intakes of vitamin A were associated with a significant increase in breast-milk concentrations of both retinol and total vitamin A. Unlike retinol, the median breast-milk β-carotene concentration was lower than the US value (57), reflecting the low provitamin A carotenoid intakes characteristic of Indonesian mothers (58). Our median breast-milk concentration of α-tocopherol unadjusted for milk fat fell within the mid-range of values in Supplemental Table 2, whereas that for γ-tocopherol was markedly higher, perhaps reflecting at least in part the frequent consumption of soya products (i.e., tempeh and tofu), a rich source of γ-tocopherol (59).

Marked discrepancies existed across countries for median breast-milk B-vitamin concentrations, especially for nicotinamide and thiamin, that may be related, at least in part, to major cereal staples consumed (60). Of the 4 water-soluble B-vitamins measured here, breast-milk concentrations of nicotinamide [the major form of niacin in breast milk (48)], and free riboflavin, secreted more efficiently in breast milk than FAD (5), were positively associated with maternal intakes. Three vitamer forms of vitamin B-1 (TMP, TPP, free thiamin), and 2 forms of vitamin B-6 (pyridoxine and pyridoxal) were measured in breast milk but none were related to maternal intakes, despite some earlier reports (61–65). Here, the prevalence of inadequate vitamin B-6 intakes was very high (∼75%), unlike thiamin and riboflavin (i.e., <40%), 2 B-vitamins supplied as fortificants in wheat flour in Indonesia (26).

Our median breast-milk vitamin B-12 concentration was within the range reported in other low-income countries where the prevalence of inadequate maternal B-12 intakes is also high (66). Nevertheless, we found no association between B-12 intakes and breast-milk concentrations, unlike some (67, 68) but not all (66) earlier studies.

The lower micronutrient intakes of these rural exclusively breastfed Indonesian infants, most notably for iron, zinc, copper, selenium, and most B-vitamins compared with the recent EFSA AIs for infants aged 0–6 mo (19), are not unexpected in view of the uncertainties surrounding the breast-milk volume (mL/d) estimate, as well as the consensus values applied for the average breast-milk micronutrient concentrations used to derive the AIs. Moreover, these same uncertain data form the basis for the additional micronutrient requirements for lactating mothers (69), and thus compromise the validity of estimates for the adequacy of their micronutrient intakes. Clearly more rigorous data are needed to generate certain values for both the average volume and micronutrient concentrations in breast milk for exclusively breastfed infants aged 0–6 mo.

Our unique study results, based on state-of-the-art methods, contribute to the limited data on intakes of micronutrients of both mothers and their exclusively breastfed infants in rural Indonesia. Our sample of lactating mothers (*n* = 113) is larger than all earlier studies to date (3, 36–40), generating important data on the interindividual variation in micronutrient intakes of exclusively breastfed infants aged 2–5.3 mo.

Nevertheless, because our study was cross-sectional, breast-milk micronutrient concentrations were only examined at a single time point, albeit the age of our infants covered the first 6 mo. In addition, we only investigated the relationship between breast-milk concentrations and current micronutrient intakes and not micronutrient status of the mothers or their dietary supplement use during pregnancy. We acknowledge that such information would provide a better understanding of long-term maternal micronutrient status in this disadvantaged rural setting. Moreover, as growth faltering was evident among these Indonesian infants, because the micronutrient status of their mothers was unknown, our data cannot be used to update the AIs for exclusively breastfed infants aged 0–6 mo. Finally, our results cannot be generalized to all lactating women in Sumedang district because we purposively recruited our participants from only 3 of 26 subdistricts.

In conclusion, the average breast-milk intake of exclusively breastfed infants we recorded was comparable to values reported globally. However, breast-milk concentrations of sodium, iron, zinc, selenium, and most vitamins measured differed from published values, with those for retinol, nicotinamide, and free riboflavin being positively associated with maternal intakes. On the basis of our measured milk volume and composition, infant intakes for most micronutrients fell below EFSA AIs. More research is needed to establish breast-milk micronutrient reference values in adequately nourished mothers and further explore the influence of both maternal micronutrient intakes and status on breast-milk concentrations through longitudinal studies.

## Supplementary Material

nqz047_Supplemental_File

## Notes

This work was supported by the Bill & Melinda Gates Foundation, Seattle, WA, USA.

Supplemental Tables 1 and 2 and Supplemental Figures 1 and 2 are available from the “Supplementary data” link in the online posting of the article and from the same link in the online table of contents at https://academic.oup.com/ajcn/.

Abbreviations used:

AI: adequate intake
EAR: estimated average requirement
EFSA: European Food Safety Authority
FAD: flavin adenine dinucleotide
IZiNCG: International Zinc Nutrition Consultative Group
MUAC: mid-upper arm circumference
RE: retinol equivalent
TMP: thiamin monophosphate
TPP: thiamin pyrophosphate
α-TE: α-tocopherol equivalent
