# Supplementary material for: Micronutrient intakes of lactating mothers and their association with breast milk concentrations and micronutrient adequacy of exclusively breastfed Indonesian infants
Source: Am J Clin Nutr. 2019 Jun 1;110(2):391–400. doi: 10.1093/ajcn/nqz047 (PMC6669051; doi:10.1093/ajcn/nqz047)
Supplement: nqz047_Supplemental_File [file nqz047_supplemental_file.docx]

Included in analysis (*n* = 113)

Enrolled (*n* = 121)

Excluded (*n* = 8)

- Failure to comply to exclusive breastfeeding (*n* = 7)
- Biologically implasiuble breastmilk volume (*n* = 1)

**Supplemental Figure 1**. Participant flowchart.

**Supplemental Figure 2.** Sensitivity analysis of the association between breast milk intake per kg of body weight and infant age, excluding infants with morbidity (*n* = 88). The estimated line from a regression model of breastmilk volume per kg of body weight (mL) = -1.44*(infant age (wks)) + 152.

**Supplemental Table 1.** Precision and accuracy of micronutrient analysis in breast milk^1^

|  | **Precision (pooled samples)** | | **Accuracy (SRM 1846)** | | **Certified value** | |
| --- | --- | --- | --- | --- | --- | --- |
| **Nutrient, mg/kg** | **Mean ± SD** | **%CV** | **Mean ± SD** | **%CV** | **Mean ± SD^2^** | **Recovery %** |
| **Minerals** |  |  |  |  |  |  |
| Sodium | 107 ± 2.7 | 2.5 | 2,184 ± 77.2 | 3.5 | 2310 ± 130 | 95 |
| Magnesium | 33.9 ± 0.9 | 2.7 | 508 ± 21.4 | 4.2 | 538 ± 29 | 94 |
| Phosphorus | 138 ± 3.6 | 2.6 | 2,415 ± 125 | 5.2 | 2,610 ± 150 | 93 |
| Potassium | 523 ± 10.7 | 2.1 | 7,136 ± 207 | 2.9 | 7,160 ± 380 | 100 |
| Calcium | 292 ± 8.3 | 2.9 | 3,600 ± 118 | 3.3 | 3,670 ± 200 | 98 |
| **Trace elements** |  |  |  |  |  |  |
| Iron | 0.18 ± 0.02 | 10.7 | 64.5 ± 2.8 | 4.4 | 63.1 ± 4.0 | 102 |
| Copper | 0.33 ± 0.005 | 1.5 | 5.0 ± 0.16 | 3.3 | 5.0 ± 0.27 | 99 |
| Zinc | 2.0 ± 0.03 | 1.5 | 59.5 ± 1.5 | 2.5 | 60 ± 3.2 | 99 |
| Selenium | 0.02 ± 0.0007 | 4.7 | 0.08 ± 0.006 | 6.8 | 0.08 | 103 |
|  | **Precision (pooled samples)** | |  | |  | |
|  | **Mean ± SD** | **%CV** |  |  |  |  |
| **Vitamins** |  |  |  |  |  |  |
| Retinol | 1.12 ± 0.04 | 3.3 |  |  |  |  |
| β-carotene | 0.06 ± 0.002 | 4.0 |  |  |  |  |
| α-tocopherol | 7.4 ± 0.19 | 2.6 |  |  |  |  |
| γ-tocopherol | 1.75 ± 0.12 | 6.8 |  |  |  |  |
| TPP | 6.7 ± 0.96 | 14.2 |  |  |  |  |
| TMP | 90.7 ± 8.6 | 9.5 |  |  |  |  |
| Free thiamin | 51.6 ± 1.67 | 3.2 |  |  |  |  |
| Vitamin B1 | 151 ± 7.4 | 4.9 |  |  |  |  |
| Free riboflavin | 279 ± 17.5 | 6.3 |  |  |  |  |
| FAD | 201 ± 32.7 | 16.2 |  |  |  |  |
| Vitamin B2 | 375 ± 25.1 | 6.7 |  |  |  |  |
| Nicotinamide | 151 ± 11.5 | 7.6 |  |  |  |  |
| Pantothenic acid | 5331 ± 260 | 4.9 |  |  |  |  |
| Pyridoxal | 246 ± 11.6 | 4.7 |  |  |  |  |
| Pyridoxine | 1.9 ± 0.20 | 10.9 |  |  |  |  |
| Vitamin B6 | 246 ± 11.5 | 4.7 |  |  |  |  |
| Biotin | 21.7 ± 0.82 | 3.8 |  |  |  |  |
| Cobalamin | 455 ± 35 | 7.6 |  |  |  |  |

^1^Abbreviations: FAD: flavin adenine dinucleotide; TMP: thiamin monophosphate; TPP: thiamin pyrophosphate

^2^Reference concentration values from the National Institute of Standards and Technology (NIST) (1)

**Supplemental Table 2.** Micronutrient concentration of breast milk from women of term infants aged 1-6 months, from the literature (from year 2000 onwards)^1^

| **Nutrient** | **Country^2^** | **Period of lactation** | ***n*** | **Micronutrient concentration^3^** | **Reference** |
| --- | --- | --- | --- | --- | --- |
| **Sodium** |  |  |  | **mg/L** |  |
|  | Africa  (Gambia) | 3 months  4 months  5 months | 183  186  191 | 95 (89, 101)*  91 (85, 98)*  85 (80, 90)* | Richards et al., 2010 (2) |
|  | Americas  (Brazil) | 2 months | 31 | 205 ± 156 | Mastroeni et al., 2006 (3) |
|  | Western Pacific  (Japan) | 1-3 months  3-6 months | 1160^4^  1160^4^ | 139 ± 72  107 ± 69 | Yamawaki et al., 2005 (4) |
|  | Western Pacific  (Mongolia) | 1-6 months | 66 | 138 ± 56 | Shi et al., 2011 (5) |
|  | Western Pacific  (South Korea) | 3 months  4 months  5 months | 87^5^  86^5^  66^5^ | 127 ± 55  101 ± 56  105 ± 37 | Kim et al., 2017 (6) |
|  | South-East Asia  (Indonesia) | 2-5.3 months | 113 | 122 (101, 193) | Daniels et al., 2018 (this study) |
| **Magnesium** |  |  |  | **mg/L** |  |
|  | Americas  (Brazil) | 2 months | 31 | 30 ± 5.0 | Mastroeni et al., 2006 (3) |
|  | Western Pacific  (Japan) | 1-3 months  3-6 months | 1170^4^  1170^4^ | 25 ± 7.0  27 ± 11 | Yamawaki et al., 2005 (4) |
|  | Western Pacific  (Mongolia) | 1-6 months | 66 | 37 ± 10 | Shi et al., 2011 (5) |
|  | Western Pacific  (South Korea) | 3 months  4 months  5 months | 87^5^  86^5^  66^5^ | 31 ± 7.0  30 ± 6.3  30 ± 6.8 | Kim et al., 2017 (6) |
|  | South-East Asia  (Indonesia) | 2-5.3 months | 113 | 30 (26, 36) | Daniels et al., 2018 (this study) |
| **Phosphorus** |  |  |  | **mg/L** |  |
|  | Americas  (Brazil) | 2 months | 31 | 137 ± 20 | Mastroeni et al., 2006 (3) |
|  | Western Pacific  (Japan) | 1-3 months  3-6 months | 1170^4^  1170^4^ | 156 ± 34  138 ± 37 | Yamawaki et al., 2005 (4) |
|  | Western Pacific  (Mongolia) | 1-6 months | 66 | 167 ± 29 | Shi et al., 2011 (5) |
|  | Western Pacific  (South Korea) | 3 months  4 months  5 months | 87^5^  86^5^  66^5^ | 139 ± 35  131 ± 32  133 ± 31 | Kim et al., 2017 (6) |
|  | South-East Asia  (Indonesia) | 2-5.3 months | 113 | 128 (119, 145) | Daniels et al., 2018 (this study) |
| **Potassium** |  |  |  | **mg/L** |  |
|  | Americas  (Brazil) | 2 months | 31 | 462 ± 84 | Mastroeni et al., 2006 (3) |
|  | Western Pacific  (Japan) | 1-3 months  3-6 months | 1167^4^  1167^4^ | 466 ± 83  434 ± 103 | Yamawaki et al., 2005 (4) |
|  | Western Pacific  (Mongolia) | 1-6 months | 66 | 504 ± 146 | Shi et al., 2011 (5) |
|  | Western Pacific  (South Korea) | 3 months  4 months  5 months | 87^5^  86^5^  66^5^ | 409 ± 110  366 ± 91  378 ± 88 | Kim et al., 2017 (6) |
|  | South-East Asia  (Indonesia) | 2-5.3 months | 113 | 435 (402, 499) | Daniels et al., 2018 (this study) |
| **Calcium** |  |  |  | **mg/L** |  |
|  | Americas  (Brazil) | 2 months | 31 | 250 ± 31 | Mastroeni et al., 2006 (3) |
|  | Western Pacific  (Japan) | 1-3 months  3-6 months | 1170^4^  1170^4^ | 257 ± 63  230 ± 74 | Yamawaki et al., 2005 (4) |
|  | Western Pacific  (Mongolia) | 1-6 months | 66 | 334 ± 70 | Shi et al., 2011 (5) |
|  | Western Pacific  (South Korea) | 3 months  4 months  5 months | 87^5^  86^5^  66^5^ | 282 ± 55  273 ± 68  271 ± 59 | Kim et al., 2017 (6) |
|  | South-East Asia  (Indonesia) | 2-5.3 months | 113 | 272 (247, 300) | Daniels et al., 2018 (this study) |
| **Iron** |  |  |  | **mg/L** |  |
|  | Americas  (USA) | 1-1.5 months  2.5-3 months | 30  17 | 0.5 ± 1.0  0.4 ± 0.3 | Hannan et al., 2008 (7) |
|  | Americas  (Brazil) | 2 months | 31 | 0.9 ± 0.5 | Mastroeni et al., 2006 (3) |
|  | Eastern Mediterranean (Iran) | 3-4 months | 182 | 0.9 ± 0.5 | Mahdavi et al. 2009 (8) |
|  | Western Pacific  (Japan) | 1-3 months  3-6 months | 1155^4^  1155^4^ | 1.8 ± 3.3  0.5 ± 1.4 | Yamawaki et al., 2005 (4) |
|  | Western Pacific  (Mongolia) | 1-6 months | 66 | 0.5 ± 0.2 | Shi et al., 2011 (5) |
|  | Western Pacific  (South Korea) | 3 months  4 months  5 months | 87^5^  86^5^  66^5^ | 0.4 ± 0.3  0.4 ± 0.7  0.3 ± 0.2 | Kim et al., 2017 (6) |
|  | South-East Asia  (India) | 3.5 months | 100^6^ | 0.3 | Shashiraj et al., 2006 (9) |
|  | South-East Asia  (Indonesia) | 2-5.3 months | 113 | 0.2 (0.2, 0.3) | Daniels et al., 2018 (this study) |
| **Copper** |  |  |  | **mg/L** |  |
|  | Europe  (Turkey) | 2 months | 142^7^ | 0.2 (0.2, 0.3) | Orun et al., 2012 (10) |
|  | Europe  (Poland) | 2-3 months  4-6 months | 108  86 | 0.2  0.1 | Winiarska-Mieczan et al., 2013 (11) |
|  | Europe  (Portugal) | 1 month | 19^8^ | 0.5 ± 0.1 | Almeida et al., 2008 (12) |
|  | Eastern Mediterranean (Iran) | 3-4 months | 182 | 0.5 ± 0.3 | Mahdavi et al. 2009 (8) |
|  | Western Pacific  (Japan) | 1-3 months  3-6 months | 1169^4^  1169^4^ | 0.3 ± 0.2  0.4 ± 0.3 | Yamawaki et al., 2005 (4) |
|  | Western Pacific  (Mongolia) | 1-6 months | 66 | 0.4 ± 0.2 | Shi et al., 2011 (5) |
|  | Western Pacific  (South Korea) | 3 months  4 months  5 months | 87^5^  86^5^  66^5^ | 0.3 ± 0.1  0.2 ± 0.1  0.2 ± 0.1 | Kim et al., 2017 (6) |
|  | South-East Asia  (Indonesia) | 2-5.3 months | 113 | 0.3 (0.2, 0.3) | Daniels et al., 2018 (this study) |
| **Zinc** |  |  |  | **mg/L** |  |
|  | Americas  (USA) | 1-1.5 months  2.5-3 months | 30  17 | 2.1 ± 1.4  2.0 ± 1.7 | Hannan et al., 2008 (7) |
|  | Americas  (Brazil) | 2 months | 31 | 1.5 ± 0.6 | Mastroeni et al., 2006 (3) |
|  | Europe  (Turkey) | 2 months | 142^7^ | 0.6 (0.5, 0.9) | Orun et al., 2012 (10) |
|  | Europe  (Poland) | 2-3 months  4-6 months | 108  86 | 1.8  0.8 | Winiarska-Mieczan et al., 2013 (11) |
|  | Europe  (Portugal) | 1 month | 19^8^ | 2.8 ± 1.2 | Almeida et al., 2008 (12) |
|  | Eastern Mediterranean (Iran) | 3-4 months | 182 | 1.9 ± 0.5 | Mahdavi et al. 2009 (8) |
|  | Western Pacific  (Japan) | 1-3 months  3-6 months | 1165^4^  1165^4^ | 1.8 ± 1.1  0.7 ± 0.8 | Yamawaki et al., 2005 (4) |
|  | Western Pacific  (China) | 3 months | 23 | 1.9 (0.2) | Xiang et al., 2007 (13) |
|  | Western Pacific  (Mongolia) | 1-6 months | 66 | 2.0 ± 1.0 | Shi et al., 2011 (5) |
|  | Western Pacific  (South Korea) | 3 months  4 months  5 months | 87^5^  86^5^  66^5^ | 1.1 ± 0.7  1.0 ± 0.6  0.9 ± 0.5 | Kim et al., 2017 (6) |
|  | South-East Asia  (Indonesia) | 2-5.3 months | 113 | 1.0 (0.7, 1.3) | Daniels et al., 2018 (this study) |
| **Selenium** |  |  |  | **μg/L** |  |
|  | Americas  (USA) | 1-1.5 months  2.5-3 months | 30  17 | 16 ± 4.1  16 ± 5.3 | Hannan et al., 2008 (7) |
|  | Europe  (Portugal) | 1 month | 19^8^ | 32 ± 8.3 | Almeida et al., 2008 (12) |
|  | Western Pacific  (Japan) | 1-3 months  3-6 months | 303^4^  303^4^ | 18 ± 4.0  15 ± 6.0 | Yamawaki et al., 2005 (4) |
|  | Western Pacific  (Mongolia) | 1-6 months | 66 | 15 ± 6.0 | Shi et al., 2011 (5) |
|  | South-East Asia  (Indonesia) | 2-5.3 months | 113 | 10 (8.7, 13) | Daniels et al., 2018 (this study) |
| **Retinol** |  |  |  | **μg/L** |  |
|  | Americas  (Canada) | 1 month | 60 | 80 ± 10 | Tijerina-Saenz et al., 2009 (14) |
|  | Europe  (Turkey) | 2-3 months | 46 | 815 ± 121 | Tokusoglu et al., 2009 (15) |
|  | Europe  (Czech Republic) | 3-4 months  5-6 months | 12  12 | 308 ± 252  224 ± 112 | Kasparova et al., 2012 (16) |
|  | Western Pacific  (Mongolia) | 1-6 months | 66 | 59 ± 36 | Shi et al., 2011 (5) |
|  | Western Pacific  (South Korea) | 3 months  4 months  5 months | 87^5^  86^5^  66^5^ | 405 ± 199  428 ± 210  364 ± 211 | Kim et al., 2017 (6) |
|  | South-East Asia  (Indonesia) | 2-5.3 months | 113 | 544 (391, 759) | Daniels et al., 2018 (this study) |
| **β-carotene** |  |  |  | **μg/L** |  |
|  | Americas  (USA) | 1 month  3 months | 17  17 | 56 ± 15  47 ± 13 | Song et al., 2013 (17) |
|  | South-East Asia  (Indonesia) | 2-5.3 months | 113 | 36 (31, 46) | Daniels et al., 2018 (this study) |
| **Vitamin A (retinol + β-carotene)** | | |  | **μg/L** |  |
|  | South-East Asia  (Bangladesh) | 2-4 months | 17 | 391 (314, 561) | Hampel et al., 2017 (18) |
|  | South-East Asia  (Indonesia) | 2-5.3 months | 113 | 598 (431, 805) | Daniels et al., 2018 (this study) |
| **α-tocopherol (E)** | |  |  | **μg/L** |  |
|  | Americas  (Canada) | 1 month | 60 | 2,320 ± 110 | Tijerina-Saenz et al., 2009 (14) |
|  | Europe  (Turkey) | 2-3 months | 46 | 9,840 ± 2,130 | Tokusoglu et al., 2009 (15) |
|  | Europe  (Poland) | 1 month  3 months | 27  19 | 2,920 ± 840  2,070 ± 660 | Martysiak-Zurowska et al., 2013 (19) |
|  | Europe  (Czech Republic) | 3-4 months  5-6 months | 12  12 | 3,747 ± 1,680  3,618 ± 1,508 | Kasparova et al., 2012 (16) |
|  | Europe  (Greece) | 3 months | 39 | 3,489 ± 1,809 | Antonakou et al., 2010 (20) |
|  | Western Pacific  (South Korea) | 3 months  4 months  5 months | 87^5^  86^5^  66^5^ | 2,200 ± 1,100  2,400 ± 1,500  2,200 ± 1,300 | Kim et al., 2017 (6) |
|  | South-East Asia  (Bangladesh) | 2-4 months | 17 | 4,400 (3,500, 6,100) | Hampel et al., 2017 (18) |
|  | South-East Asia  (Indonesia) | 2-5.3 months | 113 | 5,707 (4,240, 7,484) | Daniels et al., 2018 (this study) |
| **γ-tocopherol (E)** | |  |  | **μg/L** |  |
|  | Americas  (Canada) | 1 month | 60 | 460 ± 30 | Tijerina-Saenz et al., 2009 (14) |
|  | Europe  (Poland) | 1 month  3 months | 27  19 | 300 ± 140  220 ± 100 | Martysiak-Zurowska et al., 2013 (19) |
|  | Europe  (Spain) | 1 month | 15 | 500 | Quiles et al., 2009 (21) |
|  | South-East Asia  (Indonesia) | 2-5.3 months | 113 | 1,669 (1,266, 2,061) | Daniels et al., 2018 (this study) |
| **Thiamin Monophosphate (TMP)** | |  |  | **μg/L** |  |
|  | Africa  (Malawi) | 1.5 months | 59^9^ | 204 (172, 256) | Hampel et al., 2016 (22) |
|  | South-East Asia  (Indonesia) | 2-5.3 months | 113 | 76 (59, 94) | Daniels et al., 2018 (this study) |
| **Thiamin Pyrophosphate (TPP)** | |  |  | **μg/L** |  |
|  | Africa  (Malawi) | 1.5 months | 59^9^ | 7.6 (5.0, 13) | Hampel et al., 2016 (22) |
|  | South-East Asia  (Indonesia) | 2-5.3 months | 113 | 2.9 (2.1, 4.0) | Daniels et al., 2018 (this study) |
| **Free thiamin** |  |  |  | **μg/L** |  |
|  | Africa  (Malawi) | 1.5 months | 59^9^ | 25 (15, 36) | Hampel et al., 2016 (22) |
|  | South-East Asia  (Indonesia) | 2-5.3 months | 113 | 21 (14, 28) | Daniels et al., 2018 (this study) |
| **Total thiamin (B1)** | |  |  | **μg/L** |  |
|  | Africa  (Malawi) | 1.5 months | 59^9^ | 205 (159, 236) | Hampel et al., 2016 (22) |
|  | Europe  (Spain) | 1.5 months | 13^10^  38^10^ | 66 ± 16  157 ± 117 | Ortega et al., 2004 (23) |
|  | Western Pacific  (Mongolia) | 1-6 months | 66 | 61 ± 33 | Shi et al., 2011 (5) |
|  | South-East Asia (Bangladesh) | 2-4 months | 17 | 116 (102, 156) | Hampel et al., 2017 (18) |
|  | South-East Asia  (Indonesia) | 2-5.3 months | 113 | 99 (84, 121) | Daniels et al., 2018 (this study) |
| **Free riboflavin** | |  |  | **μg/L** |  |
|  | Africa  (Malawi) | 1.5 months | 59^9^ | 5.0 (2.3, 9.9) | Hampel et al., 2016 (22) |
|  | South-East Asia  (Indonesia) | 2-5.3 months | 113 | 8.6 (2.0, 17) | Daniels et al., 2018 (this study) |
| **Flavin Adenine Dinucleotide (FAD)** | |  |  | **μg/L** |  |
|  | Africa  (Malawi) | 1.5 months | 59^9^ | 176 (119, 272) | Hampel et al., 2016 (22) |
|  | South-East Asia  (Indonesia) | 2-5.3 months | 113 | 128 (100, 167) | Daniels et al., 2018 (this study) |
| **Total riboflavin (B2)** | |  |  | **μg/L** |  |
|  | Africa  (Malawi) | 1.5 months | 59^9^ | 91 (62, 146) | Hampel et al., 2016 (22) |
|  | Western Pacific  (Mongolia) | 1-6 months | 66 | 133 ± 41 | Shi et al., 2011 (5) |
|  | South-East Asia (Bangladesh) | 2-4 months | 17 | 24 (15, 41) | Hampel et al., 2017 (18) |
|  | South-East Asia  (Indonesia) | 2-5.3 months | 113 | 75 (58, 97) | Daniels et al., 2018 (this study) |
| **Niacin/Nicotinamide (B3)** | |  |  | **μg/L** |  |
|  | Western Pacific  (Mongolia) | 1-6 months | 66 | 1,772 ± 536^11^ | Shi et al., 2011 (5) |
|  | South-East Asia (Bangladesh) | 2-4 months | 17 | 219 (161, 295)^12^ | Hampel et al., 2017 (18) |
|  | South-East Asia  (Indonesia) | 2-5.3 months | 113 | 378 (227, 590) | Daniels et al., 2018 (this study) |
| **Pantothenic acid (B5)** | |  |  | **μg/L** |  |
|  | Western Pacific  (Mongolia) | 1-6 months | 66 | 2,418 ± 493 | Shi et al., 2011 (5) |
|  | South-East Asia  (Indonesia) | 2-5.3 months | 113 | 1,540 (1,175, 1,906) | Daniels et al., 2018 (this study) |
| **Pyridoxal (B6)** | |  |  | **μg/L** |  |
|  | South-East Asia (Bangladesh) | 2-4 months | 17 | 81 (67, 103) | Hampel et al., 2017 (18) |
|  | South-East Asia  (Indonesia) | 2-5.3 months | 113 | 64 (46, 79) | Daniels et al., 2018 (this study) |
| **Pyridoxine (B6)** | |  |  | **μg/L** |  |
|  | South-East Asia  (Indonesia) | 2-5.3 months | 32^13^ | 0.5 (0.2, 1.3) | Daniels et al., 2018 (this study) |
| **Total B6 (pyridoxal + pyridoxine)** | | |  | **μg/L** |  |
|  | Western Pacific  (Mongolia) | 1-6 months | 66 | 45 (32)^14^ | Shi et al., 2011 (5) |
|  | South-East Asia  (Indonesia) | 2-5.3 months | 113 | 64 (47, 79) | Daniels et al., 2018 (this study) |
| **Biotin (B7)** |  |  |  | **μg/L** |  |
|  | South-East Asia  (Indonesia) | 2-5.3 months | 113 | 18 (17, 19) | Daniels et al., 2018 (this study) |
| **Cobalamin (B12)** | |  |  | **μg/L** |  |
|  | Africa  (Kenya) | 1-4 months  4-6 months | 98  73 | 0.14 ± 0.13  0.14 ± 0.15 | Neumann et al., 2013 (24) |
|  | Africa  (Kenya) | 1-6 months | 286 | 0.15 (0.08, 0.27) | Williams et al., 2018  (25) |
|  | Americas  (Canada) | 2 months | 109^15^ | 0.61 (0.54, 0.68)* | Chebaya et al., 2017 (26) |
|  | Europe  (Denmark) | 4 months | 25 | 0.39 (0.19, 0.94) | Greibe et al., 2013 (27) |
|  | Western Pacific  (Cambodia) | 1-6 months | 59 | 0.43 (0.35, 0.51)* | Chebaya et al., 2017 (26) |
|  | South-East Asia (Bangladesh) | 2-4 months | 17 | 0.24 (0.12, 0.30) | Hampel et al., 2017 (18) |
|  | South-East Asia  (Indonesia) | 2-5.3 months | 113 | 0.28 (0.24, 0.34) | Daniels et al., 2018 (this study) |

^1^Excluded data from the first month postpartum due to higher breast milk concentrations of most micronutrients during this period, this time period was not assessed in our study

^2^Countries grouped by WHO regions: African Region, Region of the Americas, South-East Asia Region, European Region, Eastern Mediterranean Region, and Western Pacific Region (28)

^3^Values are mean ± SD, or geometric mean*/median (95% CI)

^4^Sample included unknown number of infants born <2.5 kg and/or mothers using supplements (unknown number and type)

^5^50% of the total sample took supplements (unknown type)

^6^Included anemic and non-anemic women

^7^*n* = 17 were pre-term, *n* = 9 were <2.5 kg; 36-88% took an iron and/or vitamin supplement during and/or post pregnancy

^8^86% of the total sample had full term infants

^9^Only used results from the initial breast milk sample of the Control group of infected HIV mothers

^10^Grouped by maternal thiamin intake in the 3^rd^ trimester of pregnancy (where *n* = 13 had intakes less than recommended; *n* = 38 had intakes greater than recommended)

^11^Reported as niacin (unknown components)

^12^Reported as niacin (vitamin B3, nicotinamide)

^13^Only 32 participants had pyridoxine present in breast milk

^14^Unknown what components made up vitamin B6 in this study

^15^Full sample received prenatal multivitamin (containing 12 μg vitamin B12) from 13-22 wks gestation, until 8 wks postpartum

**References**

1. National Institute of Standards & Technology. Standard Reference Material 1846 - Infant Formula. 2007:1–10.

2. Richards AA, Darboe MK, Tilling K, Smith GD, Prentice AM, Lawlor DA. Breast milk sodium content in rural Gambian women: between- and within-women variation in the first 6 months after delivery. Paediatric and Perinatal Epidemiology. Blackwell Publishing Ltd; 2010;24:255–61.

3. Mastroeni SSBS, Okada IA, Rondó PHC, Duran MC, Paiva AA, Neto JM. Concentrations of Fe, K, Na, Ca, P, Zn and Mg in Maternal Colostrum and Mature Milk. Journal of Tropical Pediatrics. 2006;52:272–5.

4. Yamawaki N, Yamada M, Kan-no T, Kojima T, Kaneko T, Yonekubo A. Macronutrient, mineral and trace element composition of breast milk from Japanese women. J Trace Elem Med Biol. 2005;19:171–81.

5. Shi Y-D, Sun G-Q, Zhang Z-G, Deng X, Kang X-H, Liu Z-D, Ma Y, Sheng Q-H. The chemical composition of human milk from Inner Mongolia of China. Food Chemistry. Elsevier Ltd; 2011;127:1193–8.

6. Kim H, Jung B-M, Lee B-N, Kim Y-J, Jung JA, Chang N. Retinol, α-tocopherol, and selected minerals in breast milk of lactating women with full-term infants in South Korea. Nutr Res Pract. 2017;11:64–6.

7. Hannan MA, Faraji B, Tanguma J, Longoria N, Rodriguez RC. Maternal Milk Concentration of Zinc, Iron, Selenium, and Iodine and Its Relationship to Dietary Intakes. Biol Trace Elem Res. 3rd ed. Humana Press Inc; 2008;127:6–15.

8. Mahdavi R, Nikniaz L, Gayemmagami SJ. Association Between Zinc, Copper, and Iron Concentrations in Breast Milk and Growth of Healthy Infants in Tabriz, Iran. Biol Trace Elem Res. 11 ed. Humana Press Inc; 2009;135:174–81.

9. Shashiraj, Faridi MMA, Singh O, Rusia U. Mother's iron status, breastmilk iron and lactoferrin – are they related? Eur J Clin Nutr. Nature Publishing Group; 2006;60:903–8.

10. Orun E, Yalcin SS, Aykut O, Orhan G, Morgil GK. Zinc and Copper Concentrations in Breastmilk at the Second Month of Lactation. Indian Pediatrics. 2012;49:133–5.

11. Winiarska-Mieczan A. Cadmium, Lead, Copper and Zinc in Breast Milk in Poland. Biol Trace Elem Res. Springer US; 2013;157:36–44.

12. Almeida AA, Lopes CMPV, Silva AMS, Barrado E. Trace elements in human milk: Correlation with blood levels, inter-element correlations and changes in concentration during the first month of lactation. J Trace Elem Med Biol. 2008;22:196–205.

13. Xiang M, Harbige LS, Zetterström R. Breast milk levels of zinc and ω-6 polyunsaturated fatty acids and growth of healthy Chinese infants. Acta Paediatrica. 2007;96:387–90.

14. Tijerina-Sáenz A, Innis SM, Kitts DD. Antioxidant capacity of human milk and its association with vitamins A and E and fatty acid composition. Acta Paediatrica. Blackwell Publishing Ltd; 2009;98:1793–8.

15. Tokuşoğlu Ö, Tansuğ N, Akşit S, Dinç G, Kasirga E, Özcan C. Retinol and α-tocopherol concentrations in breast milk of Turkish lactating mothers under different socio-economic status. International Journal of Food Sciences and Nutrition. 2009;59:166–74.

16. Kašparová M, Plíšek J, Solichová D, Krčmová L, Kučerová B, Hronek M, Solich P. Rapid sample preparation procedure for determination of retinol and α-tocopherol in human breast milk. Talanta. Elsevier B.V; 2012;93:147–52.

17. Song BJ, Douni ZE, Ferruzzi MG. Assessment of phytochemical content in human milk during different stages of lactation. Nutrition. Elsevier Inc; 2013;29:195–202.

18. Hampel D, Shahab-Ferdows S, Islam MM, Peerson JM, Allen LH. Vitamin Concentrations in Human Milk Vary with Time within Feed, Circadian Rhythm, and Single-Dose Supplementation. J Nutr. 2017;147:603–11.

19. Martysiak-Żurowska D, Szlagatys-Sidorkiewicz A, Zagierski M. Concentrations of alpha- and gamma-tocopherols in human breast milk during the first months of lactation and in infant formulas. Maternal & Child Nutrition. Blackwell Publishing Ltd; 2013;9:473–82.

20. Antonakou A, Chiou A, Andrikopoulos NK, Bakoula C, Matalas A-L. Breast milk tocopherol content during the first six months in exclusively breastfeeding Greek women. Eur J Nutr. 4 ed. Springer-Verlag; 2010;50:195–202.

21. Quiles JL, Ochoa JJ, Ramirez-Tortosa MC, Linde J, Bompadre S, Battino M, Narbona E, Maldonado J, Mataix J. Coenzyme Q concentration and total antioxidant capacity of human milk at different stages of lactation in mothers of preterm and full-term infants. Free Radical Research. 2009;40:199–206.

22. Hampel D, Shahab-Ferdows S, Adair LS, Bentley ME, Flax VL, Jamieson DJ, Ellington SR, Tegha G, Chasela CS, Kamwendo D, et al. Thiamin and Riboflavin in Human Milk: Effects of Lipid-Based Nutrient Supplementation and Stage of Lactation on Vitamer Secretion and Contributions to Total Vitamin Content. Mukhopadhyay P, editor. PLoS ONE. 2016;11:e0149479–14.

23. Ortega RM, Martínez RM, Andrés P, Marín-Arias L, López-Sobaler AM. Thiamin status during the third trimester of pregnancy and its influence on thiamin concentrations in transition and mature breast milk. Br J Nutr. Cambridge University Press; 2004;92:129–35.

24. Neumann CG, Oace SM, Chaparro MP, Herman D, Drorbaugh N, Bwibo NO. Low Vitamin B12 Intake during Pregnancy and Lactation and Low Breastmilk Vitamin B12 Content in Rural Kenyan Women Consuming Predominantly Maize Diets. Food and Nutrition Bulletin. 2013;34:151–9.

25. Williams AM, Stewart CP, Shahab-Ferdows S, Hampel D, Kiprotich M, Achando B, Lin A, Null CA, Allen LH, Chantry CJ. Infant Serum and Maternal Milk Vitamin B-12 Are Positively Correlated in Kenyan Infant-Mother Dyads at 1–6 Months Postpartum, Irrespective of Infant Feeding Practice. J Nutr. 2018;148:86–93.

26. Chebaya P, Karakochuk C, March K, Chen N, Stamm R, Kroeun H, Sophonneary P, Borath M, Shahab-Ferdows S, Hampel D, et al. Correlations between Maternal, Breast Milk, and Infant Vitamin B12 Concentrations among Mother–Infant Dyads in Vancouver, Canada and Prey Veng, Cambodia: An Exploratory Analysis. Nutrients. Multidisciplinary Digital Publishing Institute; 2017;9:270–10.

27. Greibe E, Lildballe DL, Streym S, Vestergaard P, Rejnmark L, Mosekilde L, Nexo E. Cobalamin and haptocorrin in human milk and cobalamin-related variables in mother and child: a 9-mo longitudinal study. Am J Clin Nutr. 2013;98:389–95.

28. WHO | WHO regional offices. WHO [Internet]. World Health Organization; 2018. Available from: www.who.int/about/regions/en/
